# Exploratory Study on the Improvement of Jump Performance Through Exposure to Schumann Frequencies

**DOI:** 10.7759/cureus.92038

**Published:** 2025-09-11

**Authors:** Saliba Danho, Johana Carolina M Vega Leonel, Diego Alexander Garzon, Juan Felipe Escobar Huertas, Harold Fajardo, Wolfgang Schoellhorn

**Affiliations:** 1 Department of Movement Science and Training, Johannes Gutenberg University Mainz, Frankfurt, DEU; 2 Neurosurgery, Stanford University, San Francisco, USA; 3 Mechanical Engineering, University of Bogota, Bogota, COL; 4 Engineering/Biomedicine, Universidad Nacional de Colombia, Bogota, COL; 5 Medicine, Universidad Nacional de Colombia, Bogotá, Colombia, Bogota, COL; 6 Department of Movement Science and Training, Johannes Gutenberg University Mainz, Mainz, DEU

**Keywords:** adolescents, electromagnetic fields (emfs), electromagnetic stimulation, neuromuscular performance, performance enhancement, rehabilitation, schumann resonance, sports medicine, vertical jump / countermovement jump

## Abstract

Studies have demonstrated that being exposed to electromagnetic fields (EMFs), especially to Schumann frequencies, may affect physiological functions. This study investigated the effects of very low frequencies (Schumann - 7.83 Hz) and low intensity (1 μT) on short- and mid-term physical jump performance of male teenagers aged 12 to 15 years in Colegio Andrés Escobar in Bogotá. A group of 20 participants was divided into two groups: one exposed to the EMFs (irradiation group) and another serving as a control without additional irradiation. Over the course of eight weeks, one-hour exposure sessions took place once per week, followed by performance evaluations. The participants performed three jumps of each type (six jumps total) per session. In sum, they completed 48 jumps (24 countermovement jumps and 24 squat jumps). The results showed a statistically significant increase in jump performance for the irradiation group, with an increase of 27% for the countermovement jump and 12% for the squat jump. In comparison, the control group exhibited non-significant decreases in both types of jumps, ranging from -3 to -1%. Although this study focused on athletic jumping tasks, the results provide evidence for a possible effect of EMFs on neuromuscular performance, with potential relevance for both athletic training and medical rehabilitation.

## Introduction

Electromagnetic fields (EMFs) may affect not only physiological processes but also physical performance and sports-related functions [1]. In addition, previous research has suggested potential links to clinical outcomes, highlighting the importance of considering both performance and health-related effects. Due to the widespread use of electronic devices and 5G, EMFs have become ubiquitous. EMFs, although regulated [2], are associated with DNA damage, membrane changes, and sleep disorders [3,4]. These fields can interfere with the body’s inherent electromagnetic processes, such as neural or cardiac signalling. EMFs cover a broad spectrum of frequencies, from extremely low-frequency (ELF) bands, which overlap with brainwaves, to high-frequency bands such as 5G [5]. This study focuses on Schumann resonances - natural ELF signals between the Earth’s surface and the ionosphere. The fundamental mode is 7.83 Hz, with harmonics at 14.3, 20.8, and 27.3 Hz, and a typical field strength in the picotesla (pT) range [6]. These frequencies coincide with the brain’s alpha band, suggesting possible neurophysiological interactions [7].

Recent theories propose that Schumann frequencies may entrain biological rhythms; they may also modulate neural activity [8,9]. Some even hypothesize an evolutionary sensitivity to these ambient signals [1].

The objective of this exploratory study was to examine whether weekly exposure to amplified Schumann frequencies (~7.83 Hz, 0.5-1 µT) influences vertical jump performance in adolescent males. Specifically, we aimed to determine (1) whether countermovement and squat jump performance would improve over time in the exposed group compared to a control group and (2) whether such improvements might suggest potential applications in sports performance and rehabilitation, for example, in neuromuscular recovery or coordination training.

## Materials and methods

Participants

As the first investigation of Schumann frequencies on jump performance, a cohort of 20 adolescents was deemed appropriate to gain initial insights and ensure efficient weekly testing within the eight-week study period, given available resources and logistical constraints. Both groups had mean ages of 13.7 and 13.0 years, with no significant difference (t(16) = 1.50, p > 0.05). Due to the study’s novelty and unknown effect sizes, no a priori power analysis was conducted. Instead, non-parametric tests and effect size calculations yielded robust, distribution-independent results. The statistical approach followed established recommendations [10]. Participants were divided into two groups: Group A (n = 10), exposed to Schumann frequencies, and Group B (control; n = 10). One B participant dropped out, leaving n = 9. Both groups performed identical tasks; jump testing was conducted separately. Only male participants aged 12-15 were recruited to ensure a homogeneous developmental stage, minimize biological variability, and avoid hormonal confounders [11]. While limited to healthy adolescents, this recruitment strategy also provides methodological insights that may be relevant for future clinical and rehabilitative research.

Ethical approval

This study was conducted at Colegio Andrés Escobar in Bogotá and approved by the Ethics Committee of the Faculty of Engineering at Universidad Nacional de Colombia (Ref. B.FI.1002-714-24; Hermes Code 63742).

Study design and procedure

Over eight weeks (with a one-week break after week three), participants completed weekly testing of countermovement and squat jumps at consistent seven-day intervals. Jump height was calculated using flight time, measured with a 1-ms-resolution mat (Sportservice-Voß) and NTG 2.0 software [12]. We selected the countermovement jump (CMJ) and squat jump (SJ) because they are standardized, reliable tests with distinct mechanics: CMJ involves stretch-shortening and coordination, while SJ isolates concentric output. This enabled us to compare coordination-related versus concentric adaptations within a consistent vertical paradigm. Horizontal tasks were beyond the scope of this pilot study. No additional training was introduced beyond the standardized jump tasks; participants only continued their regular, identical school physical education classes. Participants were not randomized due to school scheduling constraints but were assigned to fixed time slots (Group A: 10:00-11:00; Group B: 11:15-12:15), which were swapped once after week three to minimize diurnal bias. To reduce expectancy effects, the antenna was concealed, both groups sat in identical positions, and participants were unaware whether the device was active or inactive, resulting in a single-blind design.

**Figure 1:**
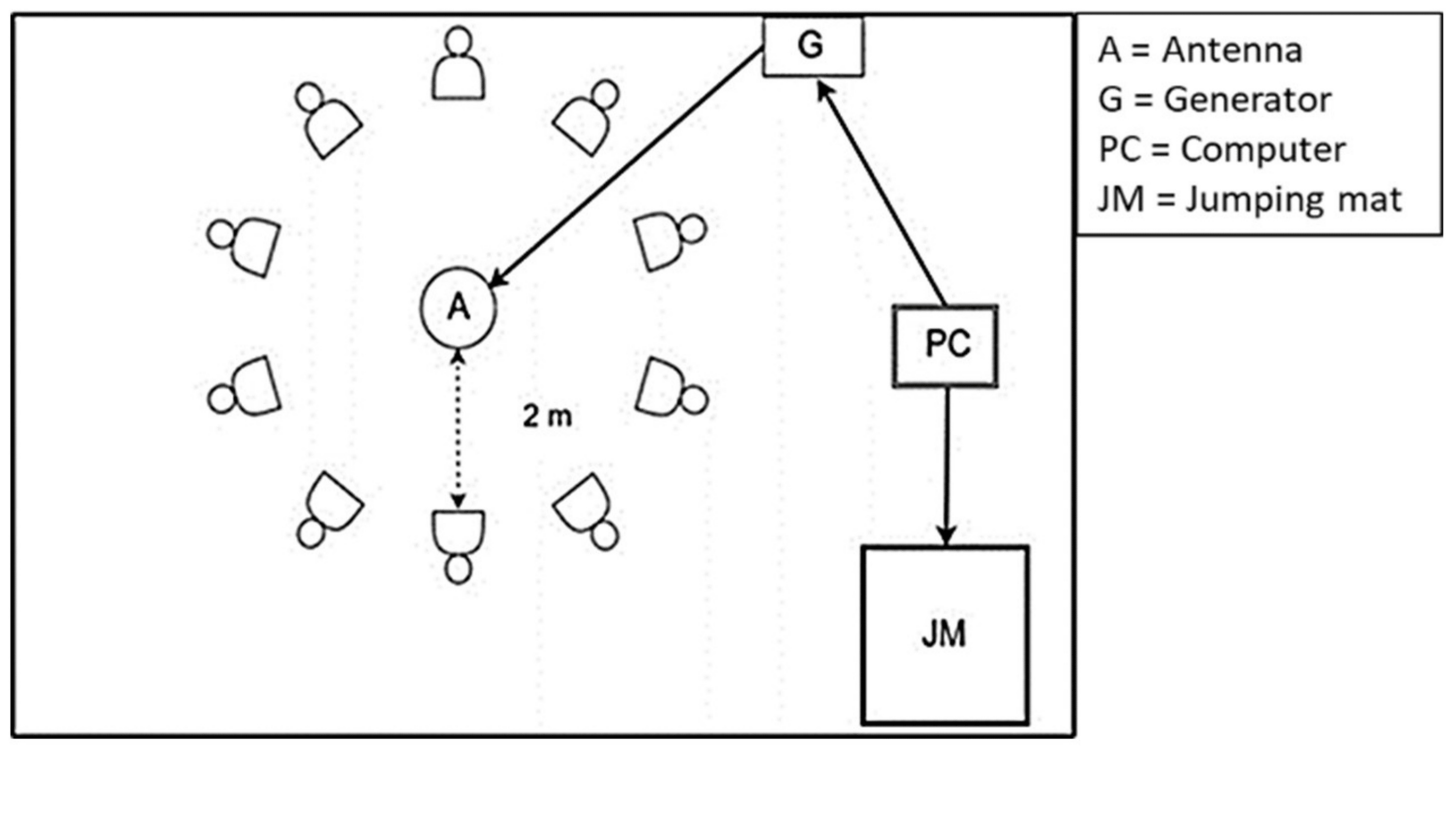
Experimental setup showing the arrangement of the generator (G), antenna (A), computer (PC), and jumping mat (JM) with participants at a 2 m distance Abbreviations: CMJ = countermovement jump; SJ = squat jump

Although the quasi-static Earth’s magnetic field weakens until noon, fluctuations (~nT) were negligible compared to the applied fluctuating 0.5-1 µT field [13]. Each weekly exposure lasted 60 minutes in the school gymnasium [14], beginning with 20 minutes seated near the antenna (on for Group A, off for Group B), followed by three squat and three countermovement jumps. EMF exposure continued throughout the full 60-minute session, including the jump phase, ensuring consistent field presence during both rest and active movement. Figure 1 illustrates the setup, including approximate distances to the antenna and jump mat.

Participants sat ~2 m from the antenna for 20 min exposure, then moved to ~2.5 m for the jumps. The overall design follows principles commonly applied in clinical pilot trials, aiming to ensure feasibility and internal validity for future translational studies.

Jump exercises

**Figure 2:**
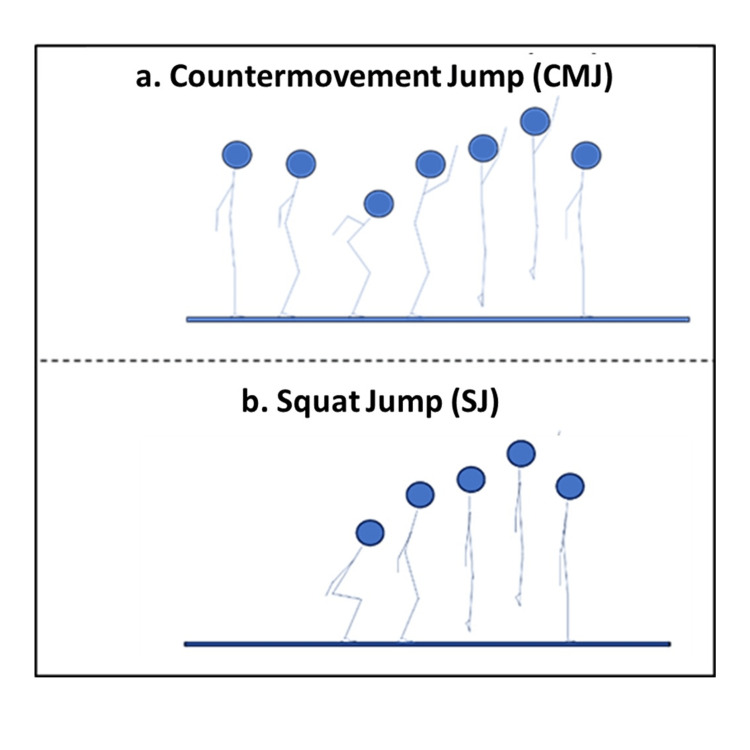
Illustration of the two jump types: countermovement jump (CMJ) with arm swing and squat jump (SJ) from 90° squat with arms extended Abbreviations: CMJ = countermovement jump; SJ = squat jump

Two standardized jump types were used (Figure 2): the countermovement jump (CMJ), a downward motion with arm swing from standing, and the squat jump (SJ), a vertical jump from a 90° squat with arms extended downward [15]. Each type was performed three times to reduce outliers.

Schumann-frequency exposure and technical validation

A 7.83 Hz electromagnetic field was generated using a commercial plasma device (Spooky SC1000 with Phanotron tube; Spooky2, China), emitting signals from 1 Hz to 3.5 MHz. The average magnetic flux density at 1 m was approximately 0.5-1 µT [6], well below the quasi-static Earth’s magnetic field (~50 µT), but far above natural Schumann levels (~1 pT). The device was calibrated prior to testing to ensure output at 7.83 Hz and its harmonics (14.3, 20.8, and 27.3 Hz). Signal stability was confirmed via long-term measurements (>1000 s) using a digital oscilloscope (Rigol MSO5104).

Electric and magnetic components were recorded using a capacitive and an inductive probe. The oscilloscope with 5 mV sensitivity recorded signals between 100 mV and ~1 V. As field strength in Tesla is not directly measured, these voltages serve as relative indicators of magnetic flux via Faraday’s law. No calibrated magnetometer or shielded environment was available, so absolute field values were approximated by modeling a theoretical dipole.

**Figure 3:**
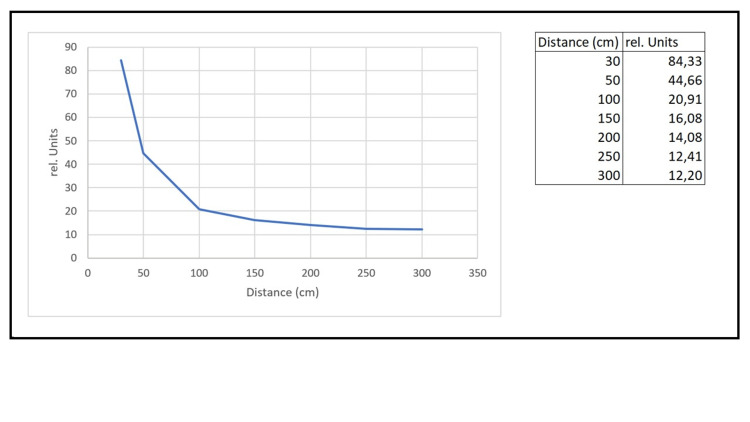
Relative field strength (normalized) vs. distance from antenna (0–300 cm); sharp drop below 100 cm, stable field beyond 100 cm The Y-axis represents relative units, and the X-axis indicates distance in centimeters. A sharp drop is visible below 100 cm, while the field strength remains stable beyond 100 cm.

A spiral Schumann antenna (Yamnis model) was used to assess spatial field distribution (see Figure 3). The measured signal followed a 1/rⁿ decay, where n depends on the antenna geometry and equals 3 for an ideal dipole. A strong signal drop-off occurred within the first meter, flattening beyond 1 m due to reflections. Figure 3 shows the relative field strength (0-3 m), measured via oscilloscope. A steep drop within the first meter followed the expected 1/rⁿ behavior (n = 3 for dipoles). Beyond 1 m, the field stabilized, likely due to reflections. Between 1 and 3 m, the field strength remained uniform (<5% angular variation), supporting internal validity. Within the 1-3 m participant range, angular variation was below 5%, indicating uniform exposure.

While voltage readings (100 mV to ~1 V) indicate field presence, these do not directly represent magnetic flux density in Tesla. Instead, they served as relative indicators, with theoretical field strengths (~0.5-1 µT) based on device specifications and modeled decay behavior.

Magnetic field strength could not be measured directly due to the absence of a Faraday cage and calibrated magnetometers. Instead, a theoretical dipole model and relative measurements based on uncalibrated probes were used. While this limits absolute precision, relative comparisons remain valid. Future studies should use calibrated equipment and shielded environments.

Earth’s magnetic field in Colombia

Bogotá’s static geomagnetic field (~35 μT) varied by <5% and was negligible [13].

By contrast, Schumann frequencies (7.83 Hz, AC) represent time-varying fields that differ fundamentally in their biophysical effects from the static geomagnetic field [16]. Alternating fields in the ELF range (extremely low frequency) show neuronal and hormonal effects in particular, which are dependent on field changes over time [1]. As the Earth's magnetic field is not an alternating field, it does not actively influence the artificially generated Schumann frequencies - it merely represents a static offset.

Statistical analysis

To adjust for age-related differences (12-15 years), jump heights were standardized by subtracting each age group’s baseline (week 0) mean. Significance was set at p < 0.05 [10]. Effect sizes were calculated using the rank-biserial correlation (r). Both raw (absolute) and standardized (relative) jump data were analyzed using consistent statistical methods.

**Figure 4:**
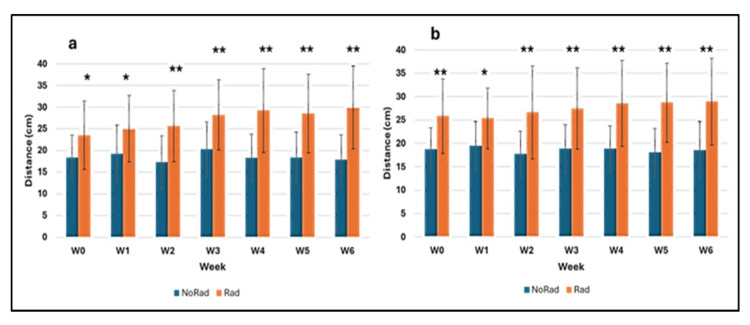
Jump heights of a) CMJ and b) SJ from week 0 to week 6 in the control group (NoRad) and the irradiated group (Rad) Differences were tested using linear regression models (with and without ORQ normalization). *p < 0.05; **p < 0.001 (statistically significant differences). Abbreviations: CMJ = countermovement jump; SJ = squat jump; NoRad = control group; Rad = irradiated group; ORQ = ordered quantile

Two linear models examined the interaction between radiation, jump type, and week. The first model analyzed raw (non-normalized) jump heights, with results shown in Figure 4. The second model used normalized data, applying the formula:

 NormJump~Radiation*TypeJump*Week (1)

An inverse transformation (1/x) was applied to the raw data to reduce skewness and stabilize variance, improving model assumptions. For standardized data, ordered quantile (ORQ) normalization mapped ranks to a normal distribution. Analyses were performed in R (v4.4.1, R Foundation, Vienna). While this technical setup is not a certified medical device, the validation approach represents an initial step toward future studies employing clinically approved EMF systems.

## Results

Figure 4 shows the jump performance of both groups from week 0 onward, regardless of age. The control group (blue) remained nearly constant throughout, while the irradiated group showed increasing differences from week 1 to week 6. The countermovement jump (CMJ) improved more than the squat jump (SJ). Overall, the irradiated group improved by 27.4% (CMJ) and 12% (SJ) compared to baseline.

**Figure 5:**
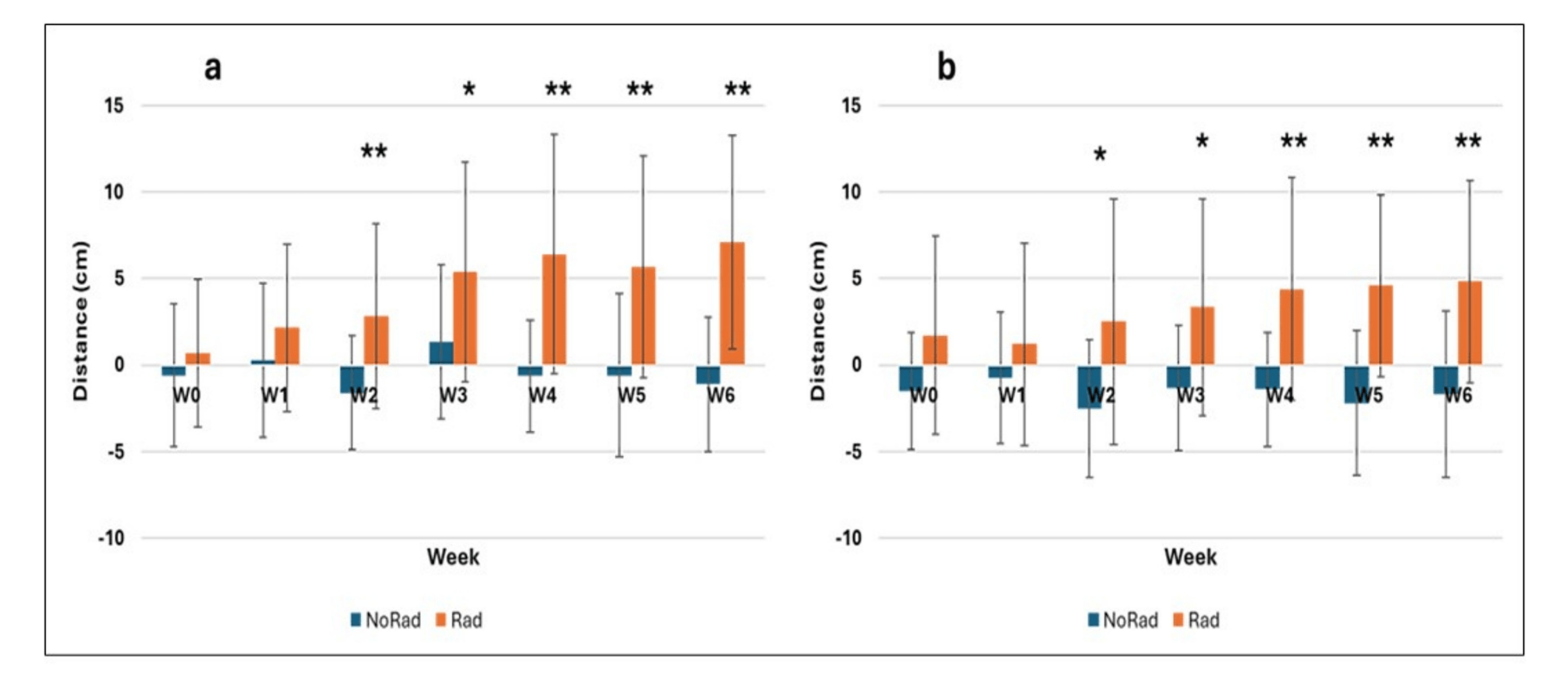
Changes in jump height from week 0 to week 6 in the control group (NoRad) and the irradiated group (Rad): a) countermovement jumps (CMJ), b) squat jumps (SJ) Group differences were tested using linear regression models (with and without ORQ normalization). Effect sizes are expressed as correlation coefficients (r). *p < 0.05; **p < 0.001 (statistically significant group differences). Abbreviations: CMJ = countermovement jump; SJ = squat jump; NoRad = control group; Rad = irradiated group; ORQ = ordered quantile

Figure 5 shows standardized changes in jump height (baseline: week 0) across all age groups (12-15 years) for both CMJ and SJ over the study period. For each group, the week 0 average was subtracted from subsequent weeks to calculate relative performance (delta). Original week 0 values are also included to show initial differences. Figure 5a (CMJ) and Figure 5b (SJ) mark statistically significant differences between groups with asterisks (*).

In the standardized data (Figure 5), jump performance in the control group (blue) remained nearly constant for both jump types. By contrast, Figure 5a shows statistically significant improvements in the irradiated group (Rad) from week 2 onward. A similar, though less pronounced, trend was observed for the squat jump (SJ; Figure 5b).

At baseline (week 0, W0), small (r = 0.109, CMJ) and medium (r = 0.291, SJ) effect sizes were found. From week 2 (W2) onward, consistently large to very large effect sizes emerged, all statistically significant (p < 0.05). For CMJ, r ranged from 0.514 (W2) to 0.778 (W6); for SJ, from 0.401 (W2) to 0.690 (W5). The strongest effects were observed at the end of the study: r = 0.778 (CMJ, W6) and r = 0.690 (SJ, W5).

**Figure 6:**
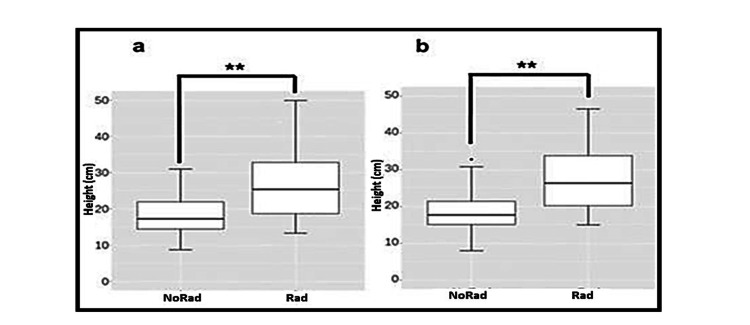
Group differences in jump height after week 6: a) CMJ, b) SJ. Boxplots display median, interquartile range, and outliers Statistical analysis was performed using linear regression models (with and without ORQ normalization). Highly significant differences between groups are marked (*p < 0.001). Abbreviations: CMJ = countermovement jump; SJ = squat jump; NoRad = control group; Rad = irradiated group; ORQ = ordered quantile

Figure 6 displays boxplots comparing jump heights at week 0 and week 6 for both groups and jump types.

The first linear model showed a significant EMF effect (p = 0.003), with a notable interaction in week 6, indicating a positive impact on the exposed group. No other time points were significant. Table 1 summarizes the interaction between radiation, jump type, and week, especially in week 6.

**Table 1:** Results of the linear regression model based on raw jump mat data Shown are estimates, standard errors, t-statistics, p-values, and 95% confidence intervals for the radiation effects and their interactions. Abbreviations: Rad = radiation (exposure) group; Estimate = regression coefficient; std_error = standard error of the estimate; statistic = t-value of the test statistic; p_value = probability value (two-sided); lower_ci / upper_ci = lower and upper bounds of the 95% confidence interval. Statistical test: ordinary least-squares linear regression (two-sided).

Term	Estimate	std_error	Statistic	p_value	lower_ci	upper_ci
Radiation: Rad	-0.012	0.004	-3.011	0.003	-0.020	-0.004
Radiation: Rad: Week 6	-0.012	0.006	-2.165	0.031	-0.024	-0.001

Applying the ORQ normalization to the standardized data (Equation 1) yielded similar results. As shown in Table 2, statistically significant interactions appeared from week 4 to week 6 in the irradiated group, indicating an effect of EMF exposure. Estimated jump height gains were 0.9 cm (week 4), 0.8 cm (week 5), and 1.1 cm (week 6) compared to the control group.

**Table 2:** Linear model interaction results based on standardized jump data Shown are estimates, standard errors, t-statistics, p-values, and 95% confidence intervals for the radiation effects across different weeks. Abbreviations: Rad = radiation (exposure) group; Week = measurement week (Week 4–6); Estimate = regression coefficient; std_error = standard error of the estimate; statistic = t-value of the test statistic; p_value = probability value (two-sided); lower_ci / upper_ci = lower and upper bounds of the 95% confidence interval; ORQ = ordered quantile (normalization method). Statistical test: ordinary least-squares linear regression (two-sided).

Term	Estimate	std_error	Statistic	p_value	lower_ci	upper_ci
Radiation: Rad: Week 4	0.892	0.334	2.669	0.008	0.236	1.548
Radiation: Rad: Week 5	0.779	0.334	2.331	0.020	0.123	1.435
Radiation: Rad: Week 6	1.144	0.334	3.422	0.001	0.488	1.800

To complement the graphical illustrations (Figures 4-6) and the regression model outputs (Tables 1-2), we also provide exact descriptive and inferential statistics. Table 3 reports group means (± SD) together with Welch’s t-tests (t, df, p) and effect sizes (r) for all weeks and both jump types, offering a transparent overview of the statistical comparisons. Together with the model outputs presented in Tables 1-2, Table 3 provides a detailed week-by-week statistical overview comparable to an ANOVA-style summary.

**Table 3:** Weekly group comparisons (Rad vs. NoRad) for countermovement jump (CMJ) and squat jump (SJ) Data are presented as mean ± SD together with Welch’s t-values (t, df), p-values, and effect sizes (r).

Week	Jump type	Rad mean (SD)	NoRad mean (SD)	t(df)	p	Effect size r
W0	CMJ	23.47 (7.86)	18.35 (5.10)	2.89 (43.8)	0.0060	0.400
W0	SJ	25.81 (7.99)	18.73 (4.60)	4.04 (40.6)	0.0002	0.536
W1	CMJ	24.95 (7.68)	19.22 (5.65)	3.18 (47.4)	0.0026	0.419
W1	SJ	25.33 (6.44)	19.49 (5.17)	3.75 (49.8)	0.0005	0.469
W2	CMJ	25.61 (8.17)	17.32 (6.06)	4.31 (47.6)	0.0001	0.530
W2	SJ	26.61 (9.89)	17.73 (4.90)	4.23 (37.2)	0.0001	0.570
W3	CMJ	28.16 (8.09)	20.27 (6.30)	4.07 (49.0)	0.0002	0.503
W3	SJ	27.44 (8.65)	18.90 (5.06)	4.49 (41.0)	0.0001	0.574
W4	CMJ	29.20 (9.66)	18.30 (5.39)	5.18 (39.8)	0.0000	0.635
W4	SJ	28.50 (9.16)	18.82 (4.88)	4.90 (38.7)	0.0000	0.619
W5	CMJ	28.47 (9.07)	18.34 (5.91)	4.94 (43.9)	0.0000	0.597
W5	SJ	28.69 (8.42)	18.03 (5.16)	5.69 (42.2)	0.0000	0.659
W6	CMJ	29.90 (9.50)	17.83 (5.75)	5.73 (41.9)	0.0000	0.663
W6	SJ	28.93 (9.25)	18.55 (6.14)	4.93 (44.5)	0.0000	0.595

## Discussion

This study set out to examine whether the physical performance of adolescents could be influenced by weekly exposure to amplified Schumann frequencies (~0.5-1 µT, 7.83 Hz + its harmonics). The exposed group showed significant improvements (CMJ: +27.4%, SJ: +12.0%), while the control group declined slightly. Increasing effect sizes over time suggest a cumulative influence. Despite being ~10⁶ times stronger than natural Schumann fields [9], no adverse effects were observed. While the results meet Fisher’s significance threshold (p < 0.05) [17], no general conclusions can be drawn. Statistical significance only indicates that further research may be warranted - not that a general rule applies. This distinction is important because broader conclusions would be based on inductive reasoning - an approach whose limitations have been acknowledged for a long time [18]. Interestingly, and contrary to this common pattern, the group with higher baseline performance in our study improved more - an effect that merits further investigation. However, it remains unclear whether the improvements reflect short-term sensitivity to ELF-EMFs or a longer-term physiological adaptation.

These improvements might be explained by one or more of the following mechanisms: Hormonal mechanisms may be involved, as neuromodulators such as irisin are associated with increased brain-derived neurotrophic factor (BDNF) expression and enhanced neuromuscular function [19]. Muscular adaptations could also play a role, with performance gains likely reflecting improved motor unit recruitment and firing rather than hypertrophy [20]. Finally, neurophysiological mechanisms may contribute, since alpha-frequency EMFs could entrain brain rhythms [8], thereby enhancing coordination through stochastic resonance [16].

This exploratory study has several limitations. First, the small sample size (n = 19) restricts statistical power and generalizability. Another limitation concerns methodological control: Although a single-blind design with concealed antennas was used, participants were not fully randomized due to school scheduling, which may have introduced bias. Second, no physiological markers (e.g., hormones, BDNF, EEG) were assessed, limiting mechanistic interpretation.

The stronger CMJ improvements may result from its higher neuromuscular demands, including eccentric loading and stretch-shortening dynamics [21,22]. In addition, evidence suggests that EMF exposure can influence neural activity patterns [21], and surface electromyography studies provide methods to assess neuromuscular activation underlying such performance adaptations [22]. However, there is also a possible coordination aspect. While the arms were actively used in the CMJ, they had to be held down during the entire jump in the SJ. This difference is important: Arm swing in CMJ introduces motor learning by demanding synchronized muscle activation and timing. This may trigger neurophysiological adaptation - especially during the eccentric downward phase - leading to greater performance gains compared to SJ, which lacks this coordination demand. This does not confound the results but suggests that EMFs may interact more strongly with coordinated, full-body movements. These neuromuscular adaptations, while demonstrated here in athletic performance, may also be relevant for rehabilitation settings, such as post-injury recovery or coordination therapy. Beyond these motor learning considerations, additional factors, such as puberty-related hormonal shifts, muscle development, and improved coordination, may also have influenced performance [19]. The role of Schumann harmonics also remains unclear. Future studies should vary frequency and intensity and apply EMG and EEG to distinguish neural from muscular mechanisms [22]. While this study focused on jumping, effects on strength or endurance are plausible. Skeletal muscle operates at 20-500 Hz [23], suggesting different resonance dynamics. Whether the higher harmonics of the Schumann frequencies, e.g., 20.8 or 27.3 Hz, resonate with these muscle frequencies demands further research. Because muscles can generate magnetic fields between 1-200 pT [24], an interaction with the produced Schumann frequencies (~0.5-1 µT) seems possible.

Recent findings also link EMF exposure to neurotransmission and circadian rhythms [25], indicating broader physiological implications. In sum, low-frequency EMFs may modulate neuromuscular performance. This perspective is consistent with established clinical applications of EMFs, such as pulsed electromagnetic fields (PEMF) used in orthopedics and transcranial magnetic stimulation (TMS) in neurology, which also aim to modulate neuromuscular and neurophysiological processes. Although our study did not assess hormonal parameters, previous research has suggested that extremely low-frequency fields, particularly at ~7.83 Hz, may exert neurohormonal effects, including modulation of BDNF or irisin expression [1]. However, such mechanisms remain speculative in the context of the current findings and warrant direct investigation in future studies. Beyond these limitations, several key areas for future research emerge: (1) effects in diverse populations (e.g., age, gender), (2) Schumann exposure parameters (duration, intensity, frequency, timing), and (3) activity types during exposure (endurance, strength, coordination, cognition). The impact on nighttime recovery and therapeutic applications also merits investigation. Future clinical studies should therefore examine whether similar effects occur in patient populations under standardized exposure protocols using certified medical devices. From a motor-learning perspective, it is noteworthy that participants with lower initial performance show greater improvements, whereas those with higher baseline performance improve less due to ceiling effects [26].

## Conclusions

As an exploratory pilot study with a small sample size and limited methodological control, the present findings provide initial evidence rather than definitive proof and should be interpreted with caution until replicated in larger, more controlled studies. This study suggests that controlled exposure to Schumann frequencies can significantly enhance physical performance, particularly in jumping. Improvements were most pronounced in countermovement jumps, likely due to enhanced neuromuscular coordination and the involvement of the stretch-shortening cycle.

The statistically significant improvements observed in the exposed group open up promising possibilities not only for athletic training and performance enhancement but also for sports medicine and rehabilitation. These effects may potentially be driven by hormonal shifts, alpha wave entrainment, or changes in neuromuscular function.

Future research should include more diverse participants, apply double-blind protocols, and examine broader capabilities such as strength and endurance. It is also important to evaluate the long-term impacts and possible risks, and to investigate potential applications in patient populations. Key EMF parameters, namely, frequency, intensity, and waveform, remain insufficiently understood, defining an important direction for future work. Together, these findings underscore the potential of EMF-based approaches for performance optimization and clinical applications.
